# Population Structure and Genetic Diversity Among Lipizzan Mare Families in Hungary Based on Microsatellite Genotyping

**DOI:** 10.3390/ani16071062

**Published:** 2026-03-31

**Authors:** Máté Kovács, Bettina Hegedűs, Sándor Mihók, Renáta Knop, Csaba Szabó, János Posta

**Affiliations:** 1 Doctoral School of Animal Science, University of Debrecen, 4032 Debrecen, Hungary; 2 Department of Animal Science, Institute of Animal Science, Biotechnology and Nature Conservation, Faculty of Agricultural and Food Sciences and Environmental Management, University of Debrecen, 4032 Debrecen, Hungary; 3 Centre for Agricultural Genomics and Biotechnology, Faculty of Agricultural and Food Sciences and Environmental Management, University of Debrecen, 4032 Debrecen, Hungary; 4 Department of Animal Nutrition and Physiology, Institute of Animal Science, Biotechnology and Nature Conservation, Faculty of Agricultural and Food Sciences and Environmental Management, University of Debrecen, 4032 Debrecen, Hungary

**Keywords:** gene conservation, breeding management, population structure, genetic differentiation, Lipizzan

## Abstract

The conservation of genetic diversity in historically structured horse breeds requires fi-ne-scale population genetic evaluation beyond conventional pedigree-based approaches. The present study assessed genetic diversity, population structure, and mare family differentiation in a Lipizzan horse population using 16 ISAG/FAO-recommended microsatellite markers. A total of 172 mares representing 29 mare families were genotyped. All loci were polymorphic, with a mean number of alleles per locus of 6.69 and a mean effective number of alleles of 3.56. The average polymorphism information content (PIC = 0.66) indicated high marker informativeness. Mean observed heterozygosity was slightly lower than expected heterozygosity, although the overall negative inbreeding coefficient suggested general heterozygote excess and low population-level inbreeding, whilst the genetic differentiation among mare families was moderate. The principal components and the STRUCTURE analysis indicated admixture among mare families. Seven private alleles were detected across six loci, highlighting the presence of rare lineage-specific variants. These findings emphasize the importance of mare family-based analyses for identifying hidden genetic structures that may not be captured by population-level averages. The integration of microsatellite-based diversity evaluation provides a robust framework for de-signing sustainable breeding and conservation strategies aimed at preserving both overall variability and rare lineage-specific genetic components.

## Simple Summary

Conserving genetic diversity is a key objective in the management of traditional horse breeds with long breeding histories. In the Lipizzan horse, mare families represent maternal lineages that may contain unique genetic variants important for the preservation of the breed. This study examined the genetic diversity and population structure of a Lipizzan population using microsatellite markers and samples from 172 mares belonging to 29 mare families. The population showed relatively high genetic diversity and low overall inbreeding, indicating a generally healthy genetic status. However, moderate genetic differences among mare families and the presence of several rare family-specific alleles were also detected. These findings demonstrate that analyses focusing on mare families can reveal hidden genetic patterns that may remain undetected in population-level assessments. Incorporating this information into breeding strategies may help preserve both the general variability of the breed and rare lineage-specific genetic variants that contribute to its long-term conservation.

## 1. Introduction

The origin of the Lipizzan breed dates back more than 400 years. In 1576, a proposal to establish a court stud was submitted to Archduke Charles II by his governor in Trieste, and the official decision was issued on 19 May 1580. The stud was founded in Lipizza (Lipica), located in the Karst region near Trieste, an area characterized by rocky and lime-stone-based terrain. The breeding objective was to develop a horse that combined the elegance and nobility of the Spanish horses with the hardiness, endurance, and modest requirements of the local Karst mares, thereby satisfying both the representational and functional demands of the Imperial court. The foundation population consisted of the best available Karst mares and, in accordance with contemporary breeding trends, imported the Spanish horses [1].

As in many other traditional breeds, the internal organization of the Lipizzan population is structured around sire lines and mare families. In contrast to sire lines, which are typically limited in number and often overrepresented in breeding schemes, mare families provide a broader and more stable representation of the genetic base of the population. Consequently, maternal lineages may capture a larger proportion of the breed’s historical genetic diversity, making them particularly relevant for conservation-oriented analyses. The preservation of these genealogical units is essential for maintaining the breed’s complete genetic heritage. The genetic structure and adaptive capacity of the population are strongly influenced by the number of active mare families and their proportional balance within the breeding population. Moreover, sustainable genetic progress can only be achieved through systematic and scientifically based breeding strategies, for which these intra-breed classification lineages provide the fundamental framework [2]. The breeding of Lipizzan horses has traditionally relied on detailed pedigree records, which allow the tracing of sire lines and mare families over several centuries. However, pedigree data primarily reflect expected genealogical relationships and do not necessarily capture the actual distribution of genetic variation within a population [3]. This limitation is particularly relevant for the Hungarian Lipizzan population, which represents a somewhat distinct component within the international population. Due to the different breeding histories and selection strategies of European stud farms, phenotypic as well as genetic differences have developed among Lipizzan populations [4]. The genetic structure of the Hungarian population is influenced by the unequal representation of mare families, which may negatively affect the distribution of genetic diversity [5]. Consequently, cryptic genetic variation within mare families may remain partially undetected, and in smaller or underrepresented lineages, there is a risk of stochastic loss of genetic diversity. Molecular genetic analyses therefore provide an important complementary approach for revealing population genetic structure and identifying lineage-specific genetic variation.

Recent studies comparing pedigree-based and genomic estimates of inbreeding have further demonstrated that pedigree information alone may underestimate realized autozygosity in small, closed populations such as the Lipizzan horse [6]. Genomic approaches based on SNP markers and runs of homozygosity (ROH) allow a more precise identification of both recent and ancient inbreeding signals, thereby complementing traditional pedigree-based monitoring. The preservation of the breed’s genetic structure, the maintenance of genetic diversity, and the protection of economically and culturally valuable traits increasingly necessitate the application of molecular genetic tools alongside conventional phenotypic and pedigree-based approaches. Among these, microsatellite markers are recognized as widely accepted and reliable instruments for population genetic analyses. Microsatellites, also known as short tandem repeats (STRs), are highly polymorphic DNA markers composed of short repeated sequence motifs distributed throughout the genome [7,8,9]. Owing to their multi-allelic nature and codominant inheritance, they provide high resolution for population genetic analyses and have long been widely used to investigate genetic variability and evolutionary processes in animal populations [10]. In equine genetics, microsatellites remain among the most widely applied molecular markers, particularly for parentage verification and lineage tracking in closed studbook populations. Their high level of polymorphism and reliable genotyping make them well suited for detecting subtle genetic differentiation among genealogical units such as sire lines and mare families. Although high-density SNP arrays have recently expanded the possibilities of genomic analyses, microsatellites continue to represent a cost-effective and widely adopted tool for evaluating genetic diversity and population structure in many breeding programs [11,12]. Microsatellite markers have been widely applied in the genetic characterization of various Equus species and breeds. Early studies on Spanish and Iberian horse populations demonstrated their effectiveness in distinguishing populations and estimating genetic variability [13]. Subsequent analyses of multiple European horse breeds, including both warmblood and cold-blood populations, confirmed the high degree of microsatellite polymorphism and the genetic differentiation among breeds [4,14,15,16]. Numerous analyses have focused on traditional and isolated populations, where microsatellite data have proven suitable for detecting inbreeding levels and assessing the risk of genetic erosion [17,18,19]. Additional studies emphasized their utility in elucidating phylogenetic relationships and historical genetic influences among populations [20,21,22,23]. More recent research has confirmed the utility of microsatellites for evaluating genetic diversity in endangered breeds and for supporting evidence-based breeding and conservation strategies [24,25,26].

While microsatellites are valuable for assessing genetic diversity and informing conservation, high-density SNP array-based studies have advanced understanding of the genetic architecture of the Lipizzan breed. Genome-wide analyses revealed clear subpopulation structure corresponding to the origin of the horses and their breeding history across European studs, while also demonstrating considerable genetic admixture between some national populations [27]. These results indicate that although historical breeding centers may show partial genetic differentiation, gene flow among subpopulations has played an important role in maintaining the overall genetic cohesion of the breed.

We hypothesized that measurable genetic structuring exists among Hungarian Lipizzan mare families. However, due to the long-term breeding practices and gene flow within the population, this structure was expected to be accompanied by substantial admixture. Furthermore, we hypothesized that mare families contribute unequally to the overall genetic diversity of the population and that certain families may harbor hidden genetic value in the form of rare, family-specific (private) alleles.

The novelty of this study lies in its fine-scale mare family-based analytical framework, which provides higher resolution insight into genetic structure compared to traditional population- or country-level analyses. This approach enables the detection of cryptic mare family-specific variation that may remain undetected in broader-scale studies and allows molecular validation of pedigree-based classification systems. Such an approach provides a robust foundation for developing evidence-based conservation and breeding strategies aimed at preserving both overall genetic variability and rare lineage-specific components over the long term. Accordingly, the objective of the present study was not merely to provide a descriptive assessment of genetic diversity, but to address the following key questions: whether mare families can be regarded as genetically distinguishable units; to what extent genetic differentiation can be detected among them; whether the population genetic architecture is better characterized as structured or predominantly admixed; and whether rare, lineage-specific genetic variants of potential conservation relevance can be identified.

## 2. Materials and Methods

### 2.1. Sampling

A total of 172 blood samples were collected from Lipizzan mares between 2023 and 2024. Blood samples were collected as part of routine mandatory veterinary health examinations conducted by a trained veterinarian. Whole blood samples were drawn into K2EDTA tubes (Becton Dickinson, Franklin Lakes, NJ, USA) and stored at −20 °C temperature until further laboratory processing. The sampled individuals represented all 29 distinct mare families within the breed currently present in the Hungarian breeding population. Mare family information was received from the official studbook of the Lipizzan breed. Individuals were selected for sampling using a randomized approach within mare families, while ensuring proportional representation of each family according to their original distribution in the breeding population. Samples were collected from 70 different breeding farms across Hungary.

### 2.2. Genomic DNA Extraction, Microsatellite Selection, PCR Amplification and Quality Control

DNA isolation from the blood samples and multiplex PCRs were performed at the Centre for Agricultural Genomics and Biotechnology, University of Debrecen. High-quality genomic DNA was isolated from whole blood samples for molecular analyses. DNA extraction was performed using a blood washing buffer (10 mM Tris-HCl, pH 7.5; 1 mM Na_2_EDTA, pH 8.0), a lysis buffer (10 mM Tris, pH 7.5; 50 mM KCl; 0.5% Tween 20) and Proteinase K enzyme (20 mg/mL; Promega, Madison, WI, USA) [28]. Samples were thoroughly resuspended and incubated at 56 °C for 90 min. Enzyme inactivation was achieved by heating the samples at 80 °C for 10 min. DNA quality and integrity were assessed by agarose gel electrophoresis, and the concentration of isolated DNA was measured using a NanoDrop1000 spectrophotometer (ThermoFisher Scientific, Waltham, MA, USA). Based on this, the genomic DNA was diluted to a final concentration of 10 ng/μL. Multiplex polymerase chain reaction (PCR) was carried out using the StockMarks™ (Wellinton, New Zealand) for Horses 17-Plex Genotyping Kit (Applied Biosystems, Foster City, CA, USA), following the manufacturer’s instructions. The kit targets 17 microsatellite loci using fluorescently labeled primer pairs (FAM, NED, PET, and VIC). However, one locus (HTG10) was excluded due to inconsistent amplification and ambiguous allele scoring. Consequently, 16 STR markers were included in the analysis. The detailed characteristics of these markers are presented in Table 1. All selected loci are included in the marker panels recommended by the International Society for Animal Genetics (ISAG) and the Food and Agriculture Organization of the United Nations (FAO) for genetic diversity studies and parentage verification. These microsatellite markers are short tandem repeats characterized by high polymorphism, codominant inheritance, and broad genome distribution, making them well suited for assessing genetic diversity and population structure in equine populations. Fragment analysis was performed by an Applied Biosystems 3130XL Genetic Analyzer (Applied Biosystems, Foster City, CA, USA) instrument. Internal sizing of PCR products was performed using the GeneScan 500 ROX size standard (Applied Biosystems, Foster City, CA, USA). Data were analyzed using Gene Mapper v5.0 software (Applied Biosystems Foster City, CA, USA).

### 2.3. Statistical Analyses

#### 2.3.1. Genetic Diversity Within and Among Mare Families

Standard parameters of genetic diversity were calculated using GenAlEx v6.51 software (Canberra, Australia) [37]. The mean number of alleles per locus (Na), the mean effective number of alleles (Ne), observed heterozygosity (Ho), expected heterozygosity (He), Hardy–Weinberg equilibrium at the locus level, Shannon’s information index (I), fixation index (F), and null allele frequency [F (Null)] were included in our study. Principal Coordinates Analysis (PCoA) was also performed in GenAlEx v6.51 (Canberra, Australia), based on a genetic distance matrix calculated for the entire population as well as among the 29 mare families, using Nei’s genetic distance. Allelic richness (AR), serving as an alternative estimator of genetic diversity, was calculated using the HP-Rare software 1.x version (Columbia, MO, USA). Wright’s F-statistics were applied to estimate F_ST_, F_IT_, and F_IS_ parameters both per locus and per mare family. The polymorphism information content (PIC) for each locus was calculated according to the equation described in [38], using Cervus v3.0.7 (Edinburgh, United Kingdom) [39].

Finally, analysis of molecular variance (AMOVA) was conducted using GenAlEx v6.51 to assess the partitioning of genetic variation under different subpopulation structures.

#### 2.3.2. Population Structure and Individual Assignment

Population structure and the relationships among the 29 mare families were investigated using STRUCTURE v2.3.3 (Stanford, CA, USA) [40]. Bayesian clustering analysis was performed under the admixture model to assign individuals to genetic clusters. Different parameter settings were tested for the burn-in period (20,000–50,000 iterations) and the number of Markov Chain Monte Carlo (MCMC) repetitions (100,000–150,000 iterations). The number of clusters (K) was set from 2 to 12 to explore potential population structure without prior assumptions, while ensuring that the upper bound exceeded the number of sampled mare families, allowing detection of potential substructure within mare families. Ten independent runs were performed for each K value. Clumpak software v1.1 (Tel Aviv, Israel) [41] was used to align and summarize replicate runs for each K value to facilitate interpretation. Graphical visualization of the results was carried out using DISTRUCT v1.0 (Stanford, CA, USA) [42]. The optimal number of clusters was determined based on the distribution of log-likelihood values LnP(K) and on the robust estimators of K implemented in StructureSelector (Berlin, Germany) [43], including MedMed K, MedMean K, MaxMed K and MaxMean K.

## 3. Results

### 3.1. Polymorphism of Microsatellite Markers

All 16 microsatellite loci analyzed were polymorphic, providing a solid basis for assessing genetic diversity and population structure within the Lipizzan population. The mean number of alleles per locus (Na) was 6.69 ± 0.39, indicating a relatively high level of genetic variability (Table 2). The highest allele numbers were observed at the ASB2 and VHL20 loci (Na = 9), whereas HTG7 exhibited the lowest number of alleles (Na = 3), suggesting more limited polymorphism at this locus. The mean effective number of alleles (Ne) was 3.56 ± 0.23, reflecting a moderately balanced allele distribution, although part of the allelic diversity was represented by low-frequency variants. The highest Ne value was recorded at HMS3 (Ne = 5.37), while the lowest values were observed at HMS1 (Ne = 2.46) and HTG6 (Ne = 2.37). Polymorphism information content (PIC) values ranged from 0.51 to 0.79, with a mean of 0.66 ± 0.02. Most markers can therefore be considered highly informative. The highest PIC value was observed at HMS3 (PIC = 0.79), followed by ASB2, ASB23, and HMS7, confirming their suitability for analyses of genetic diversity and population structure. The lowest PIC values were detected at HMS1 and HTG6 (PIC = 0.51); these loci nevertheless remained within the informative range. The mean observed heterozygosity (Ho) was 0.63 ± 0.03, while the mean expected heterozygosity (He) was 0.70 ± 0.02. Ho values varied considerably among loci (0.36–0.79). The lowest Ho values were detected at HMS3 (Ho = 0.36) and ASB17 (Ho = 0.37), where observed heterozygosity was substantially lower than expected heterozygosity, indicating heterozygote deficiency. This pattern may reflect non-random mating, population substructure or potential technical effects such as null alleles. In contrast, several loci such as AHT4, AHT5, and VHL20 showed Ho values exceeding He, suggesting heterozygote excess and low levels of inbreeding at these genomic regions. F_ST_ values varied substantially among loci. The mean F_ST_ was 0.0894 ± 0.0465, indicating moderate genetic differentiation among the analyzed mare families. Notably, HMS3 (F_ST_ = 0.5580) and ASB17 (F_ST_ = 0.4771) exhibited exceptionally high differentiation. Given the elevated null allele frequencies detected at these loci, these estimates should be interpreted with caution. The remaining loci showed low-to-moderate F_ST_ values (−0.076 to 0.065), reflecting substantial gene flow among mare families. Estimated null allele frequencies [F (Null)] were low or negative for most loci, with a mean value of 0.0391 ± 0.0202, indicating minimal overall genotyping bias. However, comparatively elevated null allele frequencies were detected at HMS3 (0.2504) and ASB17 (0.1983), which may partially explain the observed heterozygote deficiency and inflated F_ST_ values at these loci.

### 3.2. Levels of Heterozygosity and F Statistics

The dataset comprised 172 individuals representing 29 distinct mare families. Sample sizes per mare family varied substantially (N = 1–31) (Table 3). The mean number of alleles per mare family (Na) was 3.16 ± 0.23, while the mean effective number of alleles (Ne) was 2.46 ± 0.12. The highest Na values were recorded in the Presciana (Na = 5.88) and M7 (Na = 5.12) mare families, which also had the largest sample sizes (N = 31 and N = 26, respectively). This pattern likely reflects, at least in part, the influence of sample size on allelic counts. In contrast, mare families represented by a single individual (e.g., Almerina, Jadranka, Sardinia, Theodorosta, M4, M20, F1) exhibited low Na and Ne values. These reduced estimates are primarily attributable to sampling limitations rather than necessarily indicating diminished biological diversity within these mare families. The mean observed heterozygosity (Ho) across mare families was 0.61 ± 0.02, whereas the mean expected heterozygosity (He) was 0.52 ± 0.03. In the majority of mare families, Ho exceeded He, indicating an overall tendency toward heterozygote excess. The mean inbreeding coefficient (F_IS_) was −0.2917 ± 0.0781, consistent with this pattern. Strongly negative F_IS_ values (F_IS_ = −1.000) were primarily observed in mare families with very small sample sizes.

These extreme estimates are most likely methodological artifacts resulting from limited sample representation rather than evidence of biological processes. In contrast, several larger mare families, including Presciana (F_IS_ = 0.0690), M7 (F_IS_ = 0.0756), M15 (F_IS_ = 0.1153), and M16 (F_IS_ = 0.0766) exhibited slightly positive F_IS_ values, indicating moderate heterozygote deficiency. This pattern may suggest within-family mating or potential selective effects. Allelic richness (AR) values ranged within a relatively narrow interval (1.44–1.73), with a mean of 1.65 ± 0.01. The highest AR values were observed in M22 (1.73), Mozsgó Perla (1.72), and M1, M18, and F2 (approximately 1.71). Although differences among these mare families were modest, these lineages exhibited comparatively higher standardized allelic diversity within the population. Lower AR values were primarily observed in mare families represented by a single individual, where diversity estimates are likely influenced by small sample size effects. Overall, the mare family-based analysis indicates that the investigated population retains a moderate to relatively high level of genetic diversity, with diversity largely concentrated in the more numerous and well-represented mare families. The negative mean F_IS_ value and the consistent pattern of Ho exceeding He indicate an overall tendency toward heterozygote excess. This pattern is compatible with low levels of inbreeding and ongoing gene flow among mare families within the population.

Analysis of F-statistical parameters across the 16 microsatellite loci provided detailed insight into within-population inbreeding, overall heterozygosity, and genetic differentiation among subpopulations. Locus-specific F_IS_ values ranged widely from −0.3896 to 0.3629, with a mean F_IS_ of −0.1699 ± 0.0556 (Table 4). The overall negative mean FIS is consistent with a tendency toward heterozygote excess within the population. Several loci (AHT4, AHT5, CA425, HMS1, HMS6, LEX3, and VHL20) displayed distinctly negative F_IS_ values, suggesting that observed heterozygosity exceeded Hardy–Weinberg expectations at these genomic regions. In contrast, positive FIS values were observed at HMS3 (F_IS_ = 0.3629) and ASB17 (F_IS_ = 0.2880), indicating heterozygote deficiency at these loci. This pattern may result from non-random mating, population substructure (Wahlund effect), or potential selective pressures acting on these loci. F_IT_ values across loci ranged from −0.1234 to 0.6141, with a mean of 0.1262 ± 0.0527. The positive mean F_IT_ indicates a moderate heterozygote deficit at the total population level, which may reflect underlying genetic structuring or differentiation among subgroups. Notably high F_IT_ values were observed at HMS3 (0.6141) and ASB17 (0.5324), consistent with their elevated F_IS_ and F_ST_ values. These patterns highlight that these loci show marked differentiation. F_ST_ values, reflecting genetic differentiation among subpopulations, ranged from 0.1702 to 0.3943, with a mean of 0.2618 ± 0.0149, indicating moderate to relatively high genetic differentiation among the examined subgroups. The highest F_ST_ values were observed at HMS3 (0.3943), ASB17 (0.3432), ASB23 (0.3121), and ASB2 (0.3064), suggesting that these loci are particularly informative for detecting genetic structuring among populations. Conversely, the lowest F_ST_ values were observed at LEX3 (0.1702) and VHL20 (0.1908), where stronger gene flow or lower levels of differentiation may be assumed.

It should be noted that the F_ST_ values presented in Table 2 differ from those reported later in Table 4 due to differences in calculation methods. Table 2 provides locus-specific F_ST_ estimates calculated in GenAlEx, reflecting average differentiation across loci, whereas Table 4 presents Wright’s F-statistics based on the Weir and Cockerham estimator, which incorporates hierarchical population structure and variance components. These estimates therefore capture different aspects of genetic differentiation and are not directly comparable. Loci with elevated null allele frequencies (HMS3, ASB17) should be interpreted with caution.

Accordingly, the F_ST_ value (0.2618) presented in Table 4 reflects differentiation among defined mare families, whereas the estimate in Table 2 (0.0894) represents overall locus-based differentiation across the population. This difference highlights the importance of the computation method in interpreting genetic structure. Therefore, genetic parameters estimated for mare families with very small sample sizes (N ≤ 3) should be interpreted with caution and are not directly comparable to those derived from well-represented families.

### 3.3. Hardy–Weinberg Equilibrium and Locus-Specific Evaluation of Genetic Structure

Based on Hardy–Weinberg equilibrium (HWE) testing, 6 of the 16 analyzed microsatellite loci showed significant deviation from equilibrium (*p* < 0.05). These loci were ASB17, ASB2, ASB23, HMS1, HMS3, and LEX3. The remaining 10 loci did not exhibit significant deviation (*p* > 0.05), suggesting that genotype frequencies in most parts of the population are consistent with the assumption of random mating. The most pronounced deviations were observed at HMS3 (Chi^2^ = 374.796), HMS1 (Chi^2^ = 287.705), and ASB2 (Chi^2^ = 184.770), all significant at *p* = 0.000. These deviations may indicate non-random genetic processes such as population substructure (Wahlund effect) or selection acting on these loci.

### 3.4. Inbreeding Coefficient (F_IS_) and Heterozygosity

FIS values showed considerable variability, ranging from −0.198 to 0.455. For the majority of loci (AHT4, AHT5, CA425, HMS6, HTG6, HTG7, VHL20), F_IS_ values were negative, indicating heterozygote excess relative to Hardy–Weinberg expectations. In contrast, loci that significantly deviated from HWE generally exhibited positive F_IS_ values, particularly HMS3 (F_IS_ = 0.455) and ASB17 (F_IS_ = 0.404). These values indicate marked heterozygote deficiency and are consistent with the observed departure from Hardy–Weinberg equilibrium. Such patterns may reflect population substructure or non-random mating. At the LEX3 locus, a moderate but significant deviation was detected (*p* = 0.026); however, the corresponding F_IS_ value was close to zero (−0.013), indicating limited biological significance and indicating that the deviation may partly reflect sampling effects.

### 3.5. Genetic Differentiation and Distance Among Lipizzan Mare Families

Pairwise genetic differentiation among mare families was generally low-to-moderate. Locus-specific F_ST_ values ranged from 0.044 to 0.126 (Table 5), indicating measurable but limited genetic structuring among the mare families. The highest F_ST_ estimates were observed at HMS3 (0.126), AHT4 (0.120), and ASB17 (0.120), loci that also significantly deviated from Hardy–Weinberg equilibrium. These markers therefore appear particularly sensitive to underlying family-level differentiation. Conversely, AHT5 and HMS2 loci exhibited lower F_ST_ values, which is consistent with weaker differentiation and comparatively greater genetic exchange among mare families.

**Figure 1:**
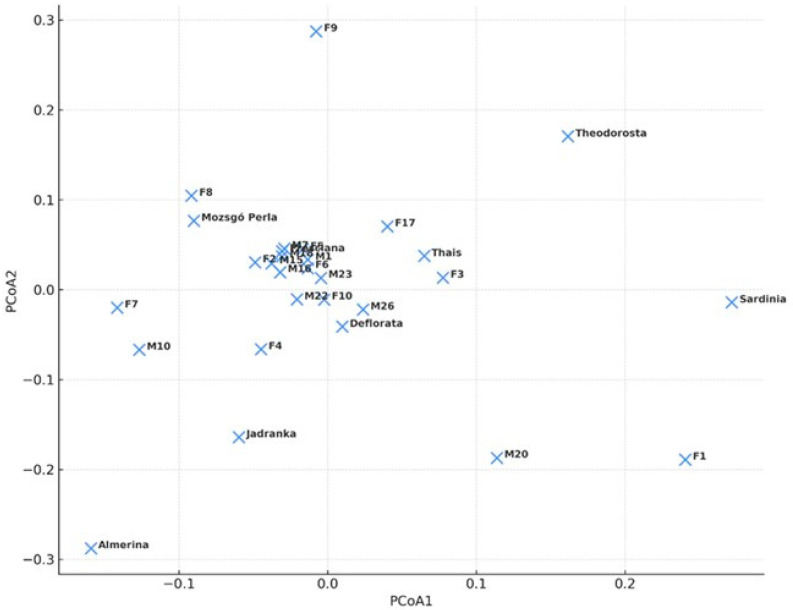
PCoA of Lipizzan mare families based on F_ST_ genetic distances calculated according to Weir and Cockerham (1984) [44].

PCoA based on F_ST_ values calculated according to Weir and Cockerham (1984) [44] was used to visualize genetic relationships among mare families (Figure 1). The first two principal coordinates explained 26.72% and 23.11% of the total variance, respectively, and separated genetically more distant mare families along the main axes of differentiation. Most mare families clustered in the central region of the coordinate space, indicating moderate differentiation and considerable genetic connectivity among them. This pattern is consistent with the previously reported low-to-moderate F_ST_ values and predominantly negative or low F_IS_ estimates. However, several mare families were positioned more distantly from the main cluster. In particular, Almerina, F1, Sardinia, Theodorosta, and M20 showed greater separation, suggesting relatively greater genetic divergence compared to the rest of the population. The absence of clearly separated clusters despite the identification of an optimal K value can be explained by the long-term breeding practices of the Lipizzan population, which include controlled gene flow among mare families and the exchange of breeding individuals across studs. As a result, the detected clusters likely represent overlapping genetic components shaped by shared ancestry and historical recombination rather than discrete biological units.

**Figure 2:**
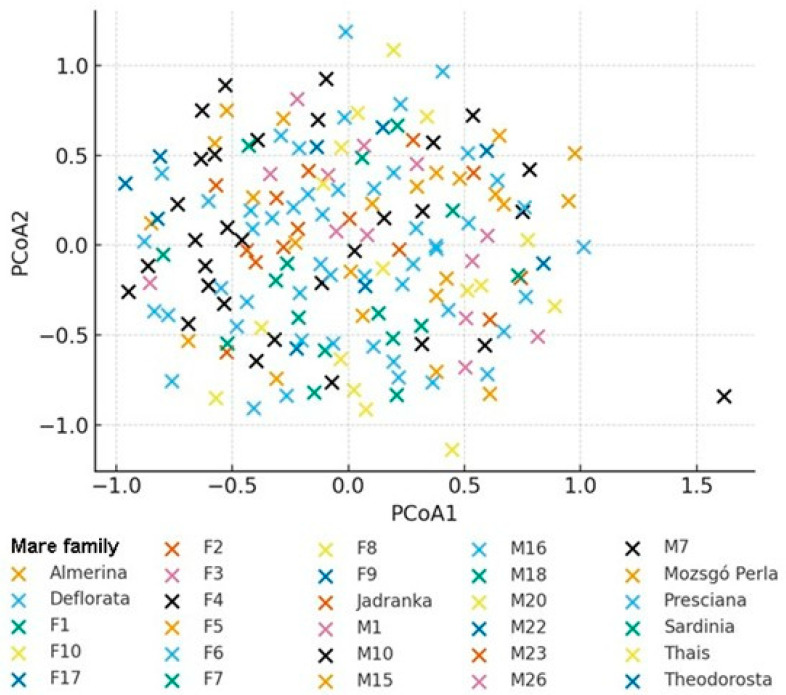
Individual-level principal coordinates analysis (PCoA) based on Nei’s genetic distance, with individuals color-coded by mare family.

**Figure 3:**
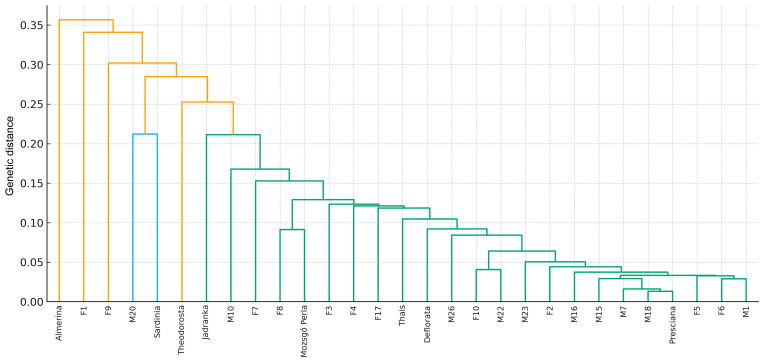
UPGMA dendrogram of Lipizzan mare families based on F_ST_ genetic distances.

As these families are represented by small sample sizes, their apparent differentiation may partly reflect sampling effects; however, historical isolation or distinct lineage origin cannot be excluded. The individual-level PCoA, based on Nei’s genetic distance and color-coded by mare family, revealed substantial overlap among families (Figure 2). The distribution of individuals was largely continuous, and no clearly separated clusters were observed. This pattern is consistent with substantial genetic admixture among mare families, suggesting that they do not function as isolated genetic units but have experienced ongoing gene flow. Individuals belonging to larger mare families (Presciana, M7, M15, and M18) were widely dispersed across the coordinate space, reflecting considerable within-family genetic heterogeneity. A small number of individuals occupied extreme (outlier) positions. These may represent unique ancestry, rare allelic combinations, or distinct within-family genetic characteristics. Such individuals may contribute disproportionately to overall population diversity. The UPGMA dendrogram constructed from F_ST_-based genetic distances was consistent with the patterns observed in the PCoA analyses (Figure 3). Most mare families clustered together at relatively low genetic distances, indicating overall genetic cohesion within the population. At higher hierarchical levels of the dendrogram, several families branched off earlier, particularly Almerina, F1, F9, M20, and, to a lesser extent, Sardinia. These families exhibited greater distances from the central cluster, consistent with their relative separation in the PCoA results.

**Figure 4:**
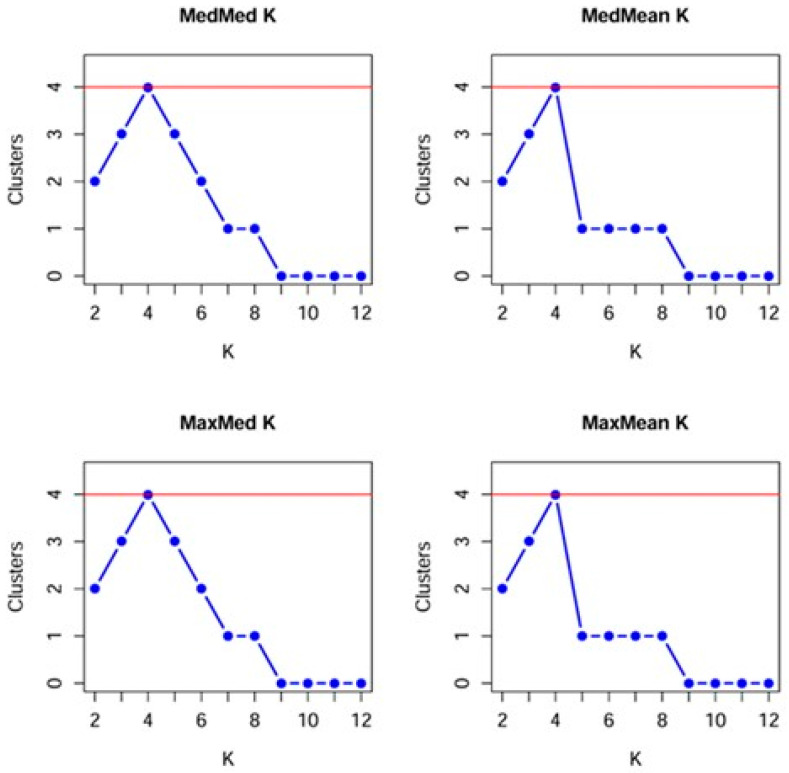
Determination of the optimal number of clusters (K = 4) using StructureSelector robust estimators.

**Figure 5:**
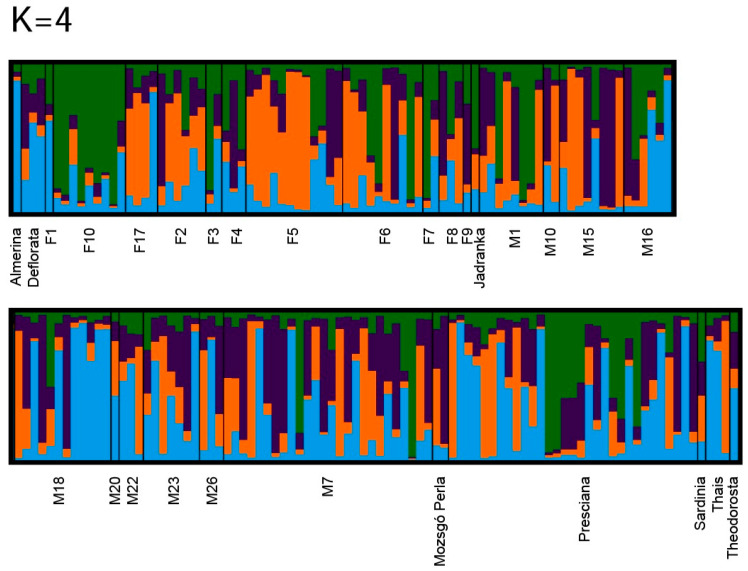
STRUCTURE bar plot for K = 4. Each bar represents an individual; colors indicate membership coefficients (Q values).

The mean LnP(K) values increased progressively from K = 2 to K = 4 and reached a plateau at approximately K = 4 (Figure 4). Beyond this point, the rate of increase slowed markedly, and the curve flattened, indicating that model fit did not improve substantially at higher K values and suggesting potential overfitting beyond this level. The plateau pattern of the LnP(K) curve is commonly interpreted as evidence that the population genetic structure can be adequately described by a limited number of partially overlapping genetic components. Different statistical implementations of the Puechmaille (2016) [43] method (MedMed K, MedMean K, MaxMed K, MaxMean K) consistently identified four clusters as the most likely number of genetic groups. Both median- and maximum-based approaches showed a clear peak at this level, whereas support declined sharply for K ≥ 6. The admixture plot corresponding to the selected cluster number (K = 4) revealed pronounced admixture throughout the entire population (Figure 5). Most individuals displayed membership across multiple clusters, and no sharply separated, homogeneous genetic groups were observed. This pattern is consistent with the strong overlap detected in the PCoA analyses, the gradual branching pattern of the UPGMA dendrogram, and the low-to-moderate F_ST_ values, all of which indicate substantial gene flow among mare families. Although certain individuals or small groups displayed dominant assignment to a particular cluster, these did not form clearly isolated genetic units within the population. Overall, the combined LnP(K) and robust K-estimator analyses indicate that the population is best described by four partially differentiated but extensively admixed genetic components. Importantly, STRUCTURE identifies statistical patterns of genetic variation rather than discrete biological populations. The strong admixture observed indicates that mare families are not reproductively isolated units but are connected through ongoing gene flow and shared breeding history. This suggests the presence of detectable structure without clear fragmentation.

### 3.6. Occurrence of Private (Unique) Alleles in the Studied Population

A total of seven private alleles were identified in the studied population. Each was detected exclusively in a single individual or within a single mare family in the analyzed sample. These alleles occurred at six different microsatellite loci (AHT4, ASB2, ASB17, HMS1, HMS6, VHL20), and their population-level frequencies were low (0.016–0.063) (Table 6). The highest frequencies (0.063) were observed for ASB2-249 in Maestoso Jázmin (M1 mare family) and ASB17–109 in Conversano XXVIII-53 (M15 mare family). Although rare at the population level, their comparatively higher frequencies within the sample suggest that they may represent family-specific genetic variants rather than recent mutations; however, this interpretation should be considered with caution given the limited sample size. The allele HMS6-165, carried by Pluto Mira (M18 mare family; f = 0.042), was also uniquely detected, further indicating the presence of family-specific genetic variants within the population. Within the Presciana mare family, several private alleles were identified (AHT4-155, ASB2-248, HMS1-180, VHL20-92), all at very low frequencies (f = 0.016–0.032). Despite its relatively large representation in the dataset, the Presciana family contributes to overall genetic diversity through internal variability and the presence of rare alleles. Furthermore, the detection of private alleles in small sample groups should be interpreted cautiously, as such variants may also arise from rare mutations or potential genotyping artifacts. Therefore, validation based on larger sample sizes and additional markers would be required to confirm their biological and conservation relevance.

## 4. Discussion

This work presents the first detailed analysis of the genetic diversity and population structure of Lipizzan mare families in Hungary. The results of the present study demonstrate that the microsatellite-based genetic diversity of the Hungarian Lipizzan population is fully consistent with values reported in international studies of the breed. The mean number of alleles per locus (Na = 6.69) and the effective number of alleles (Ne = 3.56) fall within the upper range of previously published estimates and are comparable to those reported by Achmann et al. (2004) [4] in their multi-stud analysis (MNA = 7.056), as well as to the allele numbers described by Kasarda et al. (2016) [25] for Slovak and Slovenian Lipizzan populations. These findings indicate that the allelic-level genetic variability of the investigated population has not declined relative to the broader international gene pool of the breed. The unequal distribution of sample sizes among mare families represents a limitation of the study. In particular, genetic parameters estimated for very small groups (N ≤ 3) are strongly influenced by sampling effects and are therefore not directly comparable to those derived from well-represented families. Although rarefaction was applied to standardize allelic richness, this method cannot fully compensate for extreme sampling imbalance, especially in cases where only one or a few individuals represent an entire mare family. Consequently, the interpretation of diversity parameters in such groups should be approached with caution. Although rarefaction was applied to estimate allelic richness, residual sampling bias cannot be fully excluded.

Relative to the present results, Barcaccia et al. (2013) [24] reported lower Na and Ne values in the Italian Lipizzan population, highlighting the sensitivity of diversity estimates to sampling scale and population structure. The mare family-based sampling strategy applied in the present study ensured simultaneous representation of multiple maternal lineages, thereby increasing the likelihood of detecting rare alleles. This methodological difference may partly explain the relatively high allelic diversity observed and underscores that assessment of conservation status requires evaluation not only of overall diversity but also of its distribution within the internal structure of the population.

The mean polymorphism information content (PIC = 0.66) confirms the high informativeness of the ISAG/FAO-recommended microsatellite panel. Although not all previous Lipizzan studies reported PIC values, the observed levels are consistent with the high heterozygosity previously documented in the breed [4,25]. High PIC values reflect balanced allele frequencies and strengthen the reliability of population structure inference and diversity estimation.

The discrepancy between observed heterozygosity (Ho = 0.63) and expected heterozygosity (He = 0.70) was locus-specific rather than genome-wide. Several loci exhibited heterozygote excess, whereas HMS3 and ASB17 showed pronounced heterozygote deficiency. Achmann et al. (2004) [4] described similar locus-specific deviations in Lipizzan populations and attributed them to potential null alleles or weak amplification. The elevated null allele frequencies detected at these loci in the present study are consistent with this interpretation. Importantly, such marker-specific anomalies should not be interpreted as evidence of generalized inbreeding but rather as locus-related technical or biological effects.

Interpretation of genetic parameters in small mare families should be approached with caution, as extreme F_IS_ values and low allele numbers in these cases are likely driven by sampling effects. From a breeding and conservation perspective, maintaining the more numerous and genetically diverse mare families may help preserve the bulk of population-level genetic variation. At the same time, targeted conservation measures aimed at rarer mare families could help safeguard unique, family-specific alleles. Adopting such a dual strategy may contribute to the long-term sustainability of the breed and preservation of its overall genetic diversity.

The predominantly negative F_IS_ values observed at both locus and mare family levels indicate heterozygote excess and low levels of inbreeding within the population. This pattern aligns with previous findings in the Lipizzan breed [4,25], where structured breeding management and avoidance of close inbreeding resulted in moderate or negative FIS estimates. From a conservation genetic perspective, low inbreeding levels are favorable, as they reduce the risk of inbreeding de-pression and help maintain additive genetic variance essential for long-term adaptive potential.

Genomic analyses in Lipizzan populations have similarly indicated that most autozygosity originates from distant rather than recent ancestors, suggesting that structured breeding management has generally prevented high levels of recent inbreeding [6]. While microsatellites provide high polymorphism and are well suited for detecting genetic diversity and population structure, genome-wide SNP data offer substantially higher resolution. In particular, SNP-based approaches enable the identification of runs of homozygosity, allowing the distinction between recent and ancient inbreeding events. Therefore, combining microsatellite- and SNP-based analyses would provide a more comprehensive understanding of both fine-scale structure and genome-wide diversity patterns. The F_ST_ values indicated moderate genetic differentiation among mare families and were in some cases slightly higher than differentiation estimates reported among countries or studs [4,25]. This discrepancy likely reflects differences in analytical resolution. Whereas earlier studies primarily examined geographic or stud-level subdivisions, the present analysis treated mare families as subpopulations, thereby capturing finer-scale internal structuring. Such lineage-level differentiation is expected in historically structured breeds and does not imply reproductive isolation.

Principal coordinates analysis (PCoA) and the F_ST_-based UPGMA dendrogram provided a consistent representation of the population’s genetic architecture. Most mare families clustered within a central genetic space, indicating substantial connectivity and gene flow. At the same time, several smaller or less represented families consistently occupied peripheral positions, suggesting partial differentiation. Comparable patterns of moderate differentiation embedded within extensive admixture have been reported in other Lipizzan and family-based horse populations [25,26].

The lower, densely branched portion of the dendrogram illustrates a network of closely related mare families, where short branch lengths are consistent with substantial gene flow and a shared genetic background. Taken together, the PCoA and UPGMA analyses indicate that the studied population is genetically cohesive but not entirely homogeneous (Figure 3). Most mare families are closely interconnected, reflecting moderate differentiation and substantial genetic admixture. At the same time, a small number of mare families show relative genetic separation, which may partly be explained by small sample size effects but could also reflect historical isolation or breeding practices. From a gene conservation perspective, these findings suggest that rare and genetically distinct mare families may warrant targeted management attention, while the overall high level of genetic admixture may contribute to maintaining low inbreeding and supporting long-term adaptive potential.

Bayesian clustering analysis identified four genetic components (K = 4) as the most likely structure of the population. However, these components did not form sharply separated clusters; instead, individuals exhibited pronounced admixture across components. Similar admixture patterns were observed by Kasarda et al. (2016) [25] and Yordanov et al. (2025) [26] in other structured horse populations with closed studbooks. The present results therefore support the interpretation that the Lipizzan population is genetically structured but not fragmented. The development of mare family-based genetically distinguishable units was not clearly supported by our results, as individuals from larger mare families showed reasonable genetic relationships with each other. This might suggest that breeding is not focused on mating individuals belonging to the same mare family, but rather on maximizing genetic variability within the breeding population. This configuration reflects long-term gene flow among lineages and represents a favorable balance between preservation of genealogical identity and maintenance of genome-wide diversity.

Similar patterns have also been observed in genome-wide SNP studies of Lipizzan horses, where population structure largely reflected the geographic origin of stud farms while still showing extensive admixture among subpopulations [27]. These results support the interpretation that the Lipizzan population is structured but not genetically fragmented.

The detection of seven private alleles across six loci is of relevance from a conservation standpoint. Although these alleles were present at low frequencies, they represent lineage-specific genetic variants and contribute to the overall allelic richness of the population. Barcaccia et al. (2013) [24] and other authors have emphasized that rare alleles in small or structured populations are especially vulnerable to stochastic loss through genetic drift. The extinction or underrepresentation of a single mare family carrying private alleles may therefore result in irreversible genetic erosion.

Comparable mare family-specific patterns have been reported in mitochondrial DNA studies of Lipizzan horses, where distinct haplotypes were associated with historical mare families, although some haplotypes were occasionally shared among lineages due to historical pedigree inconsistencies [45].

The presence of private alleles in both large and moderately sized families indicates that lineage-specific diversity is not restricted to demographically marginal units. This finding underscores the importance of balanced breeding strategies. Overrepresentation of certain lineages could gradually reduce effective population size and erode allelic diversity through unequal genetic contribution, even if overall heterozygosity remains apparently stable.

The occurrence of private alleles can represent an important indicator of cryptic genetic variation, particularly in breeds characterized by closed studbooks and limited effective population sizes. The low frequencies observed for most private alleles suggest that these variants may be especially susceptible to genetic drift and stochastic allele loss. Mare families harboring multiple private alleles may therefore constitute valuable genetic resources, as their conservation could contribute to the retention of rare variants and long-term population diversity. In the context of breeding program design, balanced management of such individuals and families may help reduce the risk of allele loss while maintaining overall genetic variability. Overall, the number and distribution of identified private alleles indicate that the studied population not only exhibits moderate average genetic diversity but also retains rare, family-specific genetic elements. These findings emphasize that assessment of genetic diversity should not rely exclusively on general diversity indices; consideration of private alleles can provide additional insight into the genetic value and conservation relevance of the breed. It should be noted that several private alleles were detected at very low frequencies and, in some cases, in single individuals. Such alleles may arise from rare mutations or potential genotyping artifacts, and their biological significance should be confirmed using larger sample sizes. Overall, the presence of these private alleles emphasizes the contribution of family-specific variants to the breed’s genetic diversity and illustrates the importance of preserving such rare alleles in breeding programs.

These results further demonstrate that population-level average parameters alone are insufficient for comprehensive evaluation of genetic status in historically structured breeds. A population may exhibit satisfactory overall heterozygosity while simultaneously losing internal lineage diversity. The mare family-based molecular framework applied in this study provides a more nuanced understanding of the breed’s internal genetic architecture and enables identification of lineages that disproportionately contribute to genetic variability.

Overall, the investigated Lipizzan population can be characterized as genetically diverse, moderately structured, and non-fragmented. The level of diversity corresponds to that reported in international Lipizzan populations, while the lineage-based approach applied in this study revealed additional layers of internal differentiation relevant for conservation management. Rare and partially differentiated mare families, as well as individuals carrying private alleles, represent valuable genetic resources. Their balanced conservation and management may contribute substantially to preserving both the historical genetic structure and the long-term adaptive potential of the Lipizzan breed.

## 5. Conclusions

The present study demonstrates that a fine-scale, mare family-based approach using microsatellite markers provides an effective framework for the genetic characterization of the Hungarian Lipizzan population. In an international context, the investigated population exhibits a favorable level of genetic variability, as reflected by allelic richness, effective allele number, and high marker informativeness. Although moderate genetic differentiation was detected among mare families, the population remains genetically cohesive and substantially admixed, indicating ongoing gene flow and low overall inbreeding. This structured but non-fragmented genetic architecture is consistent with long-term demographic stability and balanced breeding management. The identification of private alleles and partially differentiated mare families highlights the importance of fine-scale, mare family-based genetic evaluation. Conservation strategies that incorporate balanced lineage representation and management of rare genetic variants may help mitigate drift-driven erosion of diversity. The findings of this study provide a scientific basis for integrating molecular data into conservation-oriented breeding programs aimed at preserving both the historical genetic structure and the long-term adaptive potential of the Lipizzan breed. While microsatellites remain the gold standard for parentage control and fine-scale lineage tracking in closed studbook populations, high-density SNP arrays would enable the identification of Runs of Homozygosity, allowing the differentiation between recent and ancient inbreeding signals. Such genome-wide approaches represent an important direction for future population genomic research in equine conservation genetics. While microsatellites provide high polymorphism and are well suited for lineage-based analyses, SNP-based approaches offer genome-wide resolution. SNP data allow the detection of runs of homozygosity, enabling the distinction between recent and ancient inbreeding. The integration of SNP-based analyses would therefore complement and refine the findings of microsatellite-based studies.

## Figures and Tables

**Table 1:** Characteristics of the 16 microsatellite markers analyzed.

Locus	Chromosome	Motif	Primer Seq. 5′-3′	FOR Primer Label	Amplicon Length (bp)	Ref.
AHT4	24q14	(AC)nAT(AC)n	F: AACCGCCTGAGCAAGGAAGT R: CCCAGA-GAG-TTTACCCT	NED	144–164	[29]
AHT5	8	(GT)n	F: ACGGACACATCCCTGCCTGC R: GCAGGCTAAGGAGGCTCAGC	VIC	126–144	[29]
ASB2	15q21.3-q23	(GT)n	F: CCACTAAGTGTCGTTTCAGAAGG R: CACAACTGAG-TTCTCTGATAGG	VIC	216–250	[30]
ASB17	2p14-p15	(AC)n	F: ACCATTCAGGATCTCCACCG R: GAGGGCGGTAC-CTTT-GTACC	FAM	87–129	[30]
ASB23	3q22	(TG)n	F: GCAAGGATGAAGAGGGCAGC R: CTGGTGGGTTA-GATGAGAAGTC	NED	175–211	[31]
CA425	28q18	(GT)n	F: AGCTGCCTCGTTAATTCA R: CTCATGTCCGCTTGTCTC	FAM	226–246	[32]
HMS1	15	(TG)n	F: CATCACTCTTCATGTCTGCTTGG R: TTGACATAAATGCTTATCCTATGGC	FAM	170–186	[33]
HMS2	10	(CA)n(TC)2	F: CTTGCAGTCGAATGTGTATTAAATG R: ACGGTGG-CAACTGCCAAGGAAG	VIC	222–248	[33]
HMS3	9	(TG)2(CA)2 TC(CA)n GA(CA)5	F: CCATCCTCACTTTTTCACTTTGTT R: CCAACTCTTT-GTCACATAACAAGA	FAM	148–170	[33]
HMS6	4	(GT)n	F: GAAGCTGCCAGTATTCAACCATTG R: CTCCATCTT-GTGAAGTGTAACTCA	VIC	151–169	[33]
HMS7	1q25	(AC)2(CA)n	F: TGTTGTTGAAACATACCTTGACTGT R: CAG-GAAACTCATGTTGA-TACCATC	NED	165–185	[33]
HTG4	9	(TG)nAT(AG)5AAG(GA)5 ACAG(AGGG)3	F: CTATCTCAGTCTTGATTGCAGGAC R: CTCCCTCCCTCCCTCTGTTCTC	FAM	127–139	[34]
HTG6	15q26-q27	(TG)n	F: GTTCACTGAATGTCAAATTCTGCT R: CCTGCTT-GGAGGCTGTGATAA-GAT	FAM	84–102	[34]
HTG7	4	(GT)n	F: CCTGAAGCAGAACATCCCTCCTTG R: ATAAAGTGTCTGGG-CAGAGCTGCT	NED	118–128	[35]
LEX3	Xq	(TG)n	F: ACATCTAACCAGTGCTGAGACT R: GAAGGAAAAAAAGGAGGAAGAC	NED	142–164	[36]
VHL20	30	(TG)n	F: CAAGTCCTCTTACTTGAAGACTAG R: AACTCAGGGAGAATCTTCCTCAG	NED	87–105	[34]

**Table 2:** Genetic diversity and population genetic parameters of the 16 analyzed microsatellite loci (Na = number of alleles; Ne = effective number of alleles; PIC = polymorphism information content; Ho = observed heterozygosity; He = expected heterozygosity; I = Shannon’s information index; F_ST_ = fixation index indicating genetic differentiation; F (Null) = estimated null allele frequency).

Locus	Na	Ne	PIC	Ho	He	I	F_ST_	F (Null)
AHT4	6.00	3.55	0.67	0.77	0.72	1.41	−0.0762	−0.0319
AHT5	5.00	3.42	0.66	0.73	0.71	1.35	−0.0354	−0.0147
ASB17	6.00	3.47	0.66	0.37	0.71	1.34	0.4771	0.1983
ASB2	9.00	4.76	0.76	0.60	0.79	1.76	0.2450	0.1081
ASB23	8.00	4.75	0.76	0.61	0.79	1.76	0.2251	0.0993
CA425	6.00	2.62	0.58	0.66	0.62	1.26	−0.0753	−0.0288
HMS1	7.00	2.46	0.51	0.59	0.59	1.09	0.0050	0.0019
HMS2	8.00	3.54	0.68	0.67	0.72	1.56	0.0654	0.0273
HMS3	8.00	5.37	0.79	0.36	0.81	1.80	0.5580	0.2504
HMS6	6.00	2.72	0.56	0.65	0.63	1.18	−0.0258	−0.0100
HMS7	7.00	4.64	0.75	0.75	0.78	1.67	0.0382	0.0168
HTG4	6.00	2.84	0.61	0.62	0.65	1.33	0.0481	0.0189
HTG6	5.00	2.37	0.51	0.54	0.58	1.08	0.0649	0.0238
HTG7	3.00	2.86	0.58	0.70	0.65	1.07	−0.0735	−0.0289
LEX3	8.00	3.25	0.66	0.68	0.69	1.49	0.0119	0.0049
VHL20	9.00	4.42	0.74	0.79	0.77	1.67	−0.0220	−0.0096
Mean	6.69	3.56	0.66	0.63	0.70	1.43	0.0894	0.0391
SE	0.39	0.23	0.02	0.03	0.02	0.06	0.0465	0.0202

**Table 3:** Genetic diversity parameters of the 29 Lipizzan mare families based on 16 microsatellite loci (N = sample size; Na = mean number of alleles; Ne = effective number of alleles; Ho = observed heterozygosity; He = expected heterozygosity; AR = allelic richness; F_IS_ = inbreeding coefficient).

Mare Family *	N	Na	Ne	Ho	He	AR	F_IS_
Almerina	1	1.44	1.44	0.44	0.22	1.44	−1.0000
Deflorata	3	2.94	2.39	0.68	0.55	1.66	−0.2361
Jadranka	1	1.81	1.81	0.81	0.41	1.81	−1.0000
Presciana	31	5.88	3.41	0.64	0.69	1.70	0.0690
Sardinia	1	1.56	1.56	0.56	0.28	1.56	−1.0000
Thais	3	3.00	2.44	0.60	0.56	1.67	−0.0875
Theodorosta	1	1.69	1.69	0.69	0.34	1.69	−1.0000
M1	8	4.31	3.12	0.71	0.66	1.71	−0.0722
M4	1	1.62	1.62	0.62	0.31	1.62	−1.0000
M7	26	5.12	3.40	0.64	0.69	1.70	0.0756
M10	2	2.38	2.17	0.50	0.45	1.59	−0.1228
M15	8	4.38	3.09	0.58	0.66	1.70	0.1153
M16	6	4.06	3.03	0.59	0.64	1.70	0.0766
M18	12	4.88	3.35	0.70	0.68	1.71	−0.0197
M20	1	1.60	1.60	0.60	0.30	1.60	−1.0000
M22	3	3.19	2.77	0.65	0.61	1.73	−0.0568
M23	7	3.81	2.80	0.64	0.60	1.65	−0.0633
M26	3	3.00	2.57	0.60	0.56	1.67	−0.0807
F1	1	1.47	1.47	0.47	0.23	1.47	−1.0000
F2	6	4.06	3.03	0.69	0.65	1.71	−0.0588
F3	2	2.62	2.43	0.72	0.52	1.70	−0.3731
F4	3	2.56	2.12	0.52	0.49	1.60	−0.0563
F5	12	4.62	3.19	0.68	0.66	1.69	−0.0294
F6	10	4.81	3.15	0.63	0.65	1.68	0.0260
F7	2	2.44	2.19	0.56	0.48	1.64	−0.1803
F8	3	2.62	2.14	0.56	0.48	1.57	−0.1825
F10	9	4.06	2.68	0.58	0.58	1.62	0.0025
F17	4	2.94	2.25	0.46	0.50	1.57	0.0693
Mozsgó Perla	2	2.62	2.38	0.69	0.54	1.72	−0.2754
Mean	172	3.16	2.46	0.61	0.52	1.65	−0.2917
SE		0.23	0.12	0.02	0.03	0.01	0.0781

* Mare families with N ≤ 3 should be interpreted with caution due to limited sample size.

**Table 4:** Wright’s F-statistics for the 16 microsatellite loci (F_IS_ = inbreeding coefficient within subpopulations; F_IT_ = overall inbreeding coefficient relative to the total population; F_ST_ = genetic differentiation among subpopulations).

Locus	F_IS_	F_IT_	F_ST_
AHT4	−0.3896	−0.0596	0.2375
AHT5	−0.3380	−0.0037	0.2498
ASB17	0.2880	0.5324	0.3432
ASB2	0.0014	0.3074	0.3064
ASB23	−0.0105	0.3048	0.3121
CA425	−0.2963	0.0477	0.2654
HMS1	−0.2761	0.0993	0.2941
HMS2	−0.1747	0.0620	0.2015
HMS3	0.3629	0.6141	0.3943
HMS6	−0.2785	−0.0238	0.1992
HMS7	−0.1776	0.1559	0.2832
HTG4	−0.2312	0.0491	0.2276
HTG6	−0.2373	0.0915	0.2658
HTG7	−0.3200	0.0070	0.2478
LEX3	−0.3539	−0.1234	0.1702
VHL20	−0.2873	−0.0417	0.1908
Mean	−0.1699	0.1262	0.2618
SE	0.0556	0.0527	0.0149

**Table 5:** Results of Hardy–Weinberg equilibrium testing and population genetic parameters for the 16 microsatellite loci (Chi^2^ = chi-square statistic; P_H–W_ = *p*-value of the Hardy–Weinberg test; F_IS_ = inbreeding coefficient within subpopulations; F_ST_ = genetic differentiation among subpopulations).

Locus	Chi^2^	P_H–W_	F_IS_	F_ST_
AHT4	19.243	0.203	−0.142	0.120
AHT5	4.457	0.924	−0.123	0.044
ASB17	145.158	<0.001	0.404	0.120
ASB2	184.770	<0.001	0.079	0.112
ASB23	130.507	<0.001	0.140	0.068
CA425	9.587	0.845	−0.120	0.060
HMS1	287.705	0.000	−0.134	0.069
HMS2	13.784	0.989	0.002	0.048
HMS3	374.796	0.000	0.455	0.126
HMS6	15.829	0.394	−0.114	0.064
HMS7	27.869	0.144	−0.014	0.076
HTG4	23.205	0.080	0.018	0.096
HTG6	11.069	0.352	−0.039	0.083
HTG7	3.506	0.320	−0.198	0.064
LEX3	44.300	0.026	−0.013	0.080
VHL20	31.900	0.664	−0.065	0.066

**Table 6:** Private alleles identified in the studied Lipizzan population, including carrier individuals, associated mare families, locus, allele size, and allele frequency.

Name of the Mare	Mare Family	Locus	Allele	Allele Frequency
Maestoso Jázmin	M1	ASB2	249	0.063
Conversano XXVIII-53	M15	ASB17	109	0.063
Pluto Mira	M18	HMS6	165	0.042
Conversano Kabala	Presciana	AHT4	155	0.032
Favory Kicsi	Presciana	ASB2	248	0.016
Favory Fanni	Presciana	HMS1	180	0.016
Favory Mirza	Presciana	VHL20	92	0.016

## Data Availability

The data presented in this study are available on request from the corresponding author. The data are not publicly available due to privacy restrictions.

## References

[1] Druml T., Brem G. (2013). A fajta történelmi eredete. A Lipicai ló a Tudomány Tükrében.

[2] Mihók S. (2023). Difficulties in maintaining small horse breed populations, possible ways of decreasing the growth rate of inbreeding. Danub. Anim. Genet. Resour..

[3] Zechner P., Sölkner J., Bodó I., Druml T., Baumung R., Achmann R., Marti E., Habe F., Brem G. (2002). Analysis of diversity and population structure in the Lipizzan horse breed based on pedigree information. Liv. Prod. Sci..

[4] Achmann R., Curik I., Dovc P., Kavar T., Bodo I., Habe F., Marti E., Sölkner J., Brem G. (2004). Microsatellite diversity, population subdivision and gene flow in the Lipizzan horse. Anim. Genet..

[5] Kovács M., Mihók S. (2022). Genetic structure of the Lipizzan horse breed in Hungary through the mare families. Acta Agrar. Debreceniensis.

[6] Luštrek B., Šimon M., Turk K., Bogićević S., Potočnik K. (2025). Comparing genomic and pedigree inbreeding coefficients in the Slovenian Lipizzan horse as a case study for small closed populations. Animals.

[7] Tautz D., Renz M. (1984). Simple sequences are ubiquitous repetitive components of eukaryotic genomes. Nucleic Acids Res..

[8] Tautz D., Trick M., Dover G.A. (1986). Cryptic simplicity in DNA is a major source of genetic variation. Nature.

[9] Litt M., Luty J.A. (1989). A hypervariable microsatellite revealed by in vitro amplification of a dinucleotide repeat within the cardiac muscle actin gene. Am. J. Hum. Genet..

[10] Pérez-Gutiérrez L.M., De la Peña A., Arana P. (2008). Genetic analysis of the Hispano-Breton heavy horse. Anim. Genet..

[11] Petersen J.L., Mickelson J.R., Cothran E.G., Andersson L.S., Axelsson J., Bailey E., Bannasch D., Binns M.M., Borges A.S., Brama P. (2013). Genetic diversity in the modern horse illustrated from genome-wide SNP data. PLoS ONE.

[12] Rukavina D., Hasanbašić D., Pojskić N., Ramić J., Zahirović A., Ajanović A., Beganović K., Durmić-Pašić A. (2015). Analysis of genetic diversity among certain horse breeds from Bosnia and Herzegovina. Veterinaria.

[13] Vega-Pla J.L., Rodriguez Gallardo P.P. (1998). Investigacion biologica de la paternidad en caballos con secuencias microsatellites de AND. Med. Mil..

[14] Tozaki T., Takezaki N., Hasegawa T., Ishida N., Kurosawa M., Tomita M., Saitou N., Mukoyama H. (2003). Microsatellite variation in Japanese and Asian horses and their phylogenetic relationship using a European horse outgroup. J. Hered..

[15] Aberle K.S., Distl O. (2004). Domestication of the horse: Results based on microsatellite and mitochondrial DNA markers. Arch. Anim. Breed..

[16] Glowatzki-Mullis M.L., Muntwyler J., Pfister W., Marti E., Rieder S., Poncet P.A., Gaillard C. (2005). Genetic diversity among horse populations with a special focus on the Franches-Montagnes breed. Anim. Genet..

[17] Bruzzone A., Iamartino D., Blasi M., Pilla F. (2003). The Pentro horse: Genetic characterization by microsatellite markers. Ital. J. Anim. Sci..

[18] Szontagh A., Ban B., Bodo I., Cothran E.G., Hecker W., Jozsa C.S., Major A., Bodo I., Alderson L., Langlosi B. (2005). Genetic diversity of the Akhal-Teke horse breed in Turkmenistan based on microsatellite analysis. Conservation Genetics of Endangered Horse Breeds.

[19] Zabeck T., Nogag A., Radko A., Nogag J., Slota E. (2005). Genetic variation of Polish endangered Bilgoraj horses and two common horse breeds in microsatellite loci. J. Appl. Genet..

[20] Vega-Pla J.L., Calderon J., Rodriguez-Gallardo P.P., Alcaide B., Sereno F.T.P.S., Costa M.R., Perez-Pineda E., Martinez A.M., Delgado J.V., Rico C., Bodo I., Alderson L., Langlosi B. (2005). The retuertas horse: The “missing link” in the Iberoamerican horse breeds origin. Conservation Genetics of Endangered Horse Breeds.

[21] Vega-Pla J.L., Calderon J., Rodriguez-Gallardo P.P., Martinez A.M., Rico C. (2006). Saving feral horse populations: Does it really matter? A case study of wild horses from Doñana National Park in southern Spain. Anim. Genet..

[22] Lee S., Cho G. (2006). Parentage testing of thoroughbred horse in Korea using microsatellite DNA typing. J. Vet. Sci..

[23] Moodley Y., Baumgarten I., Harley E.H. (2006). Horse microsatellites and their amenability to comparative equid genetics. Anim. Genet..

[24] Barcaccia G., Felicetti M., Galla G., Capomaccio S., Cappelli K., Albertini E., Buttazzoni L., Pieramati C., Silvestrelli M., Verini Supplizi A. (2013). Molecular analysis of genetic diversity, population structure and inbreeding level of the Italian Lipizzan horse. Livest. Sci..

[25] Kasarda R., Moravčíková N., Kadlečík O. (2016). Spatial structure of the Lipizzan horse gene pool based on microsatellite variations analysis. AGROFOR Int. J..

[26] Yordanov G., Yordanov T., Mehandjyiski I., Radoslavov I., Salkova D., Hristov P. (2025). Population Structure and Genetic Diversity Among Shagya Arabian Horse Genealogical Lineages in Bulgaria Based on Microsatellite Genotyping. Vet. Sci..

[27] Grilz-Seger G., Druml T., Neuditschko M., Dobretsberger M., Horna M., Brem G. (2019). High-resolution population structure and runs of homozygosity reveal the genetic architecture of complex traits in the Lipizzan horse. BMC Genom..

[28] Zsolnai A., Orbán L. (1999). Accelerated separation of random complex DNA patterns in gels: Comparing the performance of discontinuous and continuous buffers. Electrophor. Int. J..

[29] Binns M.M., Holmes N.G., Holliman A., Scott A.M. (1995). The identification of polymorphic microsatellite loci in the horse and their use in thoroughbred parentage testing. Br. Vet. J..

[30] Bowling A.T., Eggleston-Stott M.L., Byrns G., Clark R.S., Dileanis S., Wictum E. (1997). Validation of microsatellite markers for routine horse parentage testing. Anim. Genet..

[31] Irvin Z., Giffard J., Brandon R., Breen M., Bell K. (1998). Equine dinucleotide repeat polymorphisms at loci ASB 21, 23, 25 and 37–43. Anim. Genet..

[32] Eggleston-Stott M.L., Valle A.D., Bautista M., Dileanis S., Wictum E., Bowling A.T. (1997). Nine equine dinucleotide repeats at microsatellite loci UCDEQ136, UCDEQ405, UCDEQ412, UCDEQ425, UCDEQ437, UCDEQ467, UCDEQ487, UCD-EQ502 and UCDEQ505. Anim. Genet..

[33] Guerin G., Bertaud M., Amigues Y. (1994). Characterization of seven new horse microsatellites: HMS1, HMS2, HMS3, HMS5, HMS6, HMS7 and HMS8. Anim. Genet..

[34] Ellegren H., Johansson M., Sandberg K., Andersson L. (1992). Cloning of highly polymorphic microsatellites in the horse. Anim. Genet..

[35] Marklund S., Ellegren H., Eriksson S., Sandberg K., Andersson L. (1994). Parentage testing and linkage analysis in the horse using a set of highly polymorphic microsatellites. Anim. Genet..

[36] Coogle L., Bailey E. (1998). Equine dinucle otide repeat lociLEX064 through LEX070. Anim. Genet..

[37] Peakall R., Smouse P.E. (2012). GenAlEx 6.5: Genetic analysis in Excel. Population genetic software for teaching and research-an update. Bioinformatics.

[38] Botstein D., White R.L., Skolnick M., Davis R.W. (1980). Construction of a genetic linkage map in man using restriction fragment length polymorphisms. Am. J. Hum. Genet..

[39] Kalinowski S.T., Taper M.L., Marshall T.C. (2007). Revising how the computer program CERVUS accommodates genotyping error increases success in paternity assignment. Mol. Ecol..

[40] Pritchard J.K., Stephens M., Donnelly P. (2000). Inference of population structure using multilocus genotype data. Genetics.

[41] Kopelman N.M., Mayzel J., Jakobsson M., Rosenberg N.A., Mayrose I. (2015). Clumpak: A program for identifying clustering modes and packaging population structure inferences across K. Mol. Ecol. Resour..

[42] Rosenberg N.A. (2004). Distruct: A program for the graphical display of population structure. Mol. Ecol. Notes.

[43] Puechmaille S.J. (2016). The program structure does not reliably recover the correct population structure when sampling is uneven: Subsampling and new estimators alleviate the problem. Mol. Ecol. Resour..

[44] Weir B.S., Cockerham C.C. (1984). Estimating F-statistics for the analysis of population structure. Evolution.

[45] Crisà A., Cardinali I., Giontella A., Silvestrelli M., Lancioni H., Buttazzoni L. (2024). A genetic make up of Italian Lipizzan horse through uniparental markers to preserve historical pedigrees. Biology.

